# The complete chloroplast genome sequence of *Melastoma candidum* (Melastomataceae)

**DOI:** 10.1080/23802359.2017.1318680

**Published:** 2017-04-24

**Authors:** Wei Lun Ng, Yacheng Cai, Wei Wu, Renchao Zhou

**Affiliations:** State Key Laboratory of Biocontrol and Guangdong Provincial Key Laboratory of Plant Resources, School of Life Sciences, Sun Yat-sen University, Guangzhou, China

**Keywords:** *Melastoma*, Melastomataceae, species, radiation, complete, chloroplast, genome, automated, assembly

## Abstract

The plant genus *Melastoma* is comprised of members estimated to have formed through recent species radiation. Natural hybridization among member species further complicates taxonomy within the genus. Herein, we report the complete chloroplast genome of *M. candidum*, assembled from partial data obtained from a parallel whole-genome Illumina paired-end sequencing effort on the species. The chloroplast genome was 156,682 bp in length, with a large single-copy (LSC) region of 86,084 bp, a small single-copy (SSC) region of 17,094 bp, separated by two inverted repeat (IR) regions of 26,752 bp each. It was predicted to contain a total of 129 genes, with an overall GC content of 37.17%. Phylogenetic analysis placed *M. candidum* in the same clade as species within the Melastomeae tribe of Melastomataceae.

The plant genus *Melastoma* comprises of species distributed mainly in the tropical and subtropical regions of Asia and Oceania (Meyer 2001). The recognized number of species remains debatable until today, with claims ranging from 22 (Meyer 2001), 80–90 (Wong 2016), to about 100 (Chen 1984) species. The diversity observed in *Melastoma* is thought to be the outcome of species radiation, estimated to have happened within the past one million years (Renner & Meyer 2001). Recent studies have also found widespread hybridization among the different species (e.g. Dai et al. 2012; Liu et al. 2014; Wong 2015), further complicating species delineation within the genus. Here, we report the complete chloroplast genome sequence of *M. candidum*, commonly found in southern China, as a resource for future studies on the taxonomy of *Melastoma*.

Sequence data used for the assembly of this chloroplast genome was extracted from the total sequencing data from a parallel whole-genome Illumina paired-end sequencing effort of an *M. candidum* individual (Wu et al., unpublished data) sampled from Wenchang, Hainan, China. The voucher specimen (MCAN-2013-HN01) is kept at the Sun Yat-sen University Herbarium (SYS). Approximately 4 Gb of paired-end (125 bp) sequence data was randomly extracted from the total sequencing output, as input into NOVOPlasty (Dierckxsens et al. 2017) to assemble the chloroplast genome. A partial chloroplast *rbc*L gene sequence of the same species (GenBank accession GQ436728) was used as the seed sequence for the seed-and-extend algorithm implemented in NOVOPlasty. The accuracy of the automated assembly was then verified by Sanger-sequencing of seven randomly chosen genes (*rps*16, *atp*F, *rpo*C2, *rpo*C1, *atp*E, *rpo*A, and *ndh*D), covering approximately 14 kbp (∼8.9% of the total chloroplast genome size), with 100% accuracy. Annotation of the chloroplast genome was performed using Verdant (McKain et al. 2017) and DOGMA (Wyman et al. 2004), then manually verified and corrected by comparison with sequences on GenBank.

The complete chloroplast genome sequence of *M. candidum* (GenBank accession KY745894) obtained in this study was 156,682 bp in length, with a large single-copy (LSC) region of 86,084 bp, a small single-copy (SSC) region of 17,094 bp, separated by two inverted repeat (IR) regions of 26,752 bp each. It was predicted to contain 129 genes, including 85 protein-coding genes, 36 tRNA genes, and 8 rRNA genes. The overall GC content was 37.17%. The *ndh*D gene had an ACG start codon, instead of the conventional AUG start codon.

For phylogenetic tree construction, the chloroplast genome of *M. candidum* was aligned with 16 other complete chloroplast genome sequences of Melastomataceae (Reginato et al., 2016) and *Eucalyptus globulus* (AY780259) as outgroup, using MAFFT v7.307 (Katoh & Standley 2013). A maximum likelihood tree (Figure 1) was then constructed using RAxML (Stamatakis, 2014). *Melastoma candidum* shared the same clade with two other genera within the Melastomeae tribe, *Petrogastra* and *Tibouchina*.

**Figure 1.**
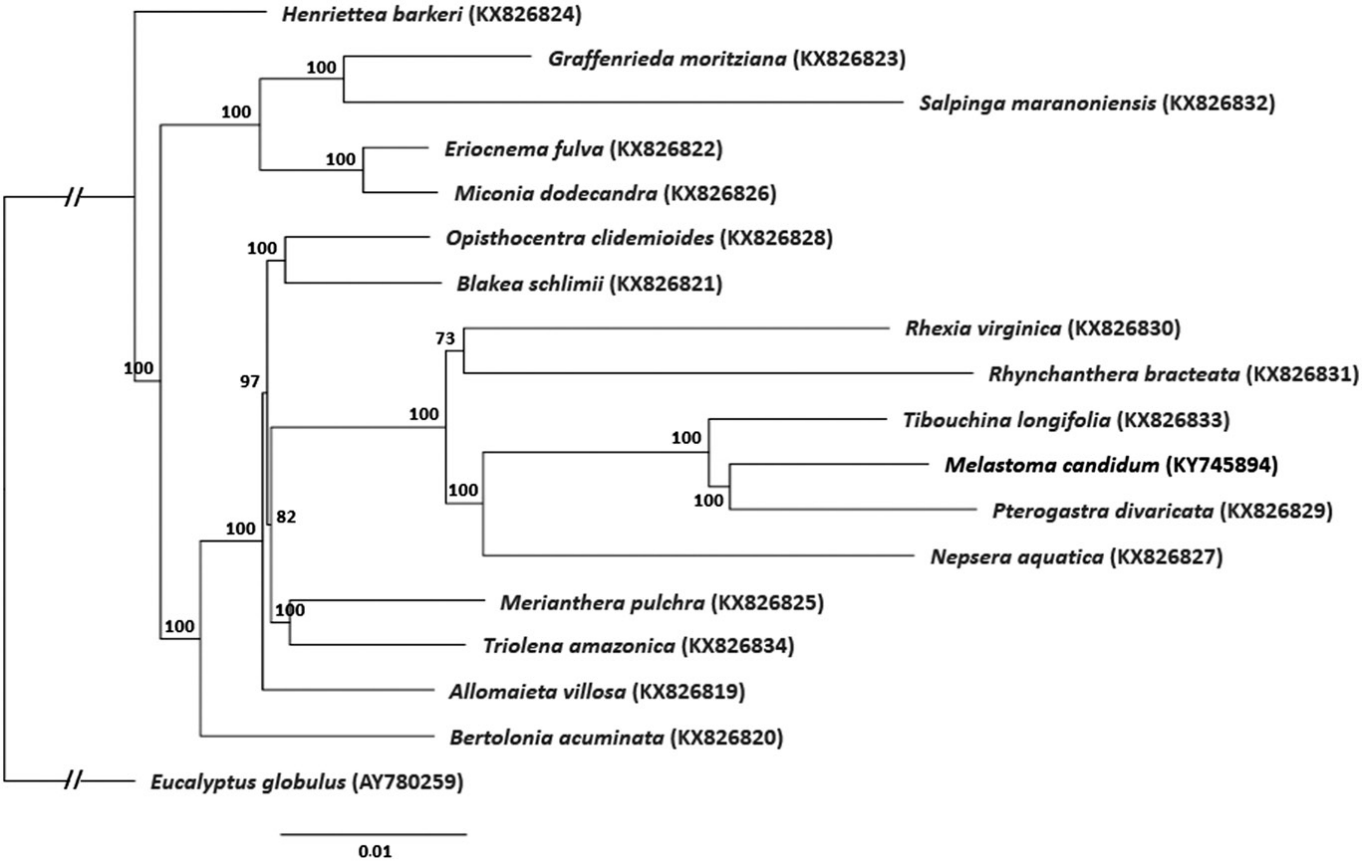
Maximum likelihood tree of Melastomataceae based on complete chloroplast genomes, with *Eucalyptus globulus* as outgroup. Bootstrap support values (based on 1000 replicates) are shown next to the nodes. Scale in substitutions per site.
